# Effect of Perioperative CEA and CA24-2 on Prognosis of Early Resectable Pancreatic Ductal Adenocarcinoma

**DOI:** 10.7150/jca.33767

**Published:** 2020-01-01

**Authors:** Xiaojie Li, Shengnan Li, Lili Liu, Jiahui Hong, Tiansuo Zhao, Chuntao Gao

**Affiliations:** 1 Department of Pancreatic Cancer, Tianjin Medical University Cancer Institute and Hospital, National Clinical Research Center for Cancer, Key Laboratory of Cancer Prevention and Therapy, Tianjin's Clinical Research Center for Cancer, Tianjin, China, 300060; 2 Department of Nutrition and Food Hygiene, School of Public Health, Tianjin Medical University, Tianjin, China, 300060

**Keywords:** Pancreatic cancer, Radical resection, CEA antigen, CA24-2 antigen, Prognosis

## Abstract

Patients with resectable pancreatic ductal adenocarcinoma (PDAC) show differential prognosis after radical resection. Currently, cancer grading and surgical criteria depend heavily on imaging and anatomical diagnosis. It's essential to set up a model with reliable prognostic factors during the perioperative period to assess prognosis in PDAC patients. In this study, 103 patients diagnosed with PDAC who underwent radical resection were recruited. The predictive value of preoperative carcinoembryonic antigen (CEA), postoperative CA24-2 and the combination of two for overall survival (OS) were evaluated. Both pre-CEA and post-CA24-2 were found to be independent prognostic factors for OS according to multivariate analyses. Kaplan-Meier analysis revealed that CEA and CA24-2 as well as the combination of two were correlated with poor OS. In addition, patients with both markers elevated have worse prognosis than patients with either pre-CEA or post-CA24-2 elevated. Thus, we concluded that the combination of CEA and CA24-2 can be used as a prognostic factor for stage I and II resectable PDAC patients.

## Introduction

Pancreatic ductal adenocarcinoma (PDAC) is the fourth leading cause of cancer mortality worldwide with a five-year survival rate of approximately 7.2% 1. Up to date, surgery is the only effective treatment option for PDAC, which can increase the 5-year survival rate by 20%-25% 2, 3. However, only 15%-20% of patients diagnosed during early cancer stage are eligible for radical surgery. Furthermore, this invasive technique does not guarantee a cure, and sometimes may lead to poor response to chemotherapy 4.

Currently, surgical guideline of PDAC still relies on imaging and tumor anatomy. Studies of postoperative PDAC prognosis have shown that clinical pathological features such as tumor size, lymph node metastasis, neurological invasion, distant metastasis, and vascular invasion may influence disease-specific survival(DSS), disease-free survival (DFS), and overall survival(OS) 5, 6, 7. Serological indicators such as LDH, ALB, ALP, NLR and CRP affect prognosis of patients with stage III and IV PDAC characterized by distant metastasis and local progression 8. While serum CA19-9, CA12-5 and CEA have been reported as markers of prognosis for PDAC at all stages 9. Moreover, TNM Classification of Malignant Tumors may also affect treatment plan and prognosis of patients 10. Overall there are no reliable biological markers to guide adjuvant therapy and determine prognosis of PDAC. In this study, our retrospective cohort analysis investigated clinical data, laboratory tests and pathological diagnosis before and after PDAC resection. We aim to identify possible markers related to PDAC radical resection in order to provide a basis for clinical evaluation of patient prognosis.

## Materials and Methods

### Patients

103 patients diagnosed with PDAC and underwent radical resection in Tianjin Medical University Cancer Institute and Hospital from 2011 to 2015 were recruited for our study. Eligibility for radical resection was evaluated according to NCCN guideline 11. Pancreaticoduodenectomy or spleen and distal pancreatectomy were performed based on tumor location and growth. Patients underwent admission assessments including chest X-ray, abdominal CT or MR, and baseline observations were recorded. Postoperative tissue biopsy confirmed pancreatic ductal adenocarcinoma diagnosis.

All patients underwent postoperative adjuvant chemotherapy within 1 or 2 months after surgery. We conducted regular telephone follow-up of all enrolled patients. The exclusion criteria for this study included: 1. Preoperative assessment was not found, but intraoperative pathology diagnosed patients with distant metastases. 2. Non-R0 resection of the patient. 3. Patients who died within 1 month after surgery. 4. Patients who did not undergo standard adjuvant chemotherapy after surgery. 5. Patients who were unwilling to undergo research or unable to follow up. This study has passed the ethical review of the Ethics Committee of the Tianjin Medical University Cancer Institute and Hospital.

### Histopathological examination

Tumor tissues used for diagnostics were all collected during surgery. The histopathological criteria used in this study included: tumor size, tumor location, carcinoma cell embolism, lymph node metastasis, distant metastasis, perineural invasion, pancreatic capsule invasion, and presence of R0 resection. TNM staging of PDAC were determined based on histopathology in accordance with the American Joint Commission on Cancer (AJCC) 11.

### Laboratory testing

Serum CA19-9, CEA, CA24-2, LDH, ALP, TBIL, DBIL, ALB, and NLR were collected within 7 days before surgery. ALB was collected again 7 days after surgery. CA19-9, CEA and CA24-2 were collected 30 days after surgery. The cut-off values were defined as the upper limits of normal CA19-9, CEA, CA24-2, LDH, ALP, TBIL, DBIL, ALB, and the lower limits of normal ALB. We set the cutoff value of NLR to 5.

### Study design and statistical analysis

All statistical analyses were performed using SPSS software (version20.0). Data between groups were compared using Pearson χ^2^ and Fisher's exact, and the inter-group OS was compared using non-parametric statistics. Univariate COX regression was used to screen for potential prognostic factors, and p value ≤0.20 was used for multivariate COX regression to calculate HR and determine independent prognostic factors. Survival analysis was performed using the Kaplan-Meier method. Survival period is the interval between the day of surgery and death or loss of follow-up. Statistical significance was defined as p value ≤0.05.

## Results

### Patient characteristics

This study included 103 patients, 65 males (63.1%) and 38 females (36.9%), with PDAC who were admitted to the Tianjin Medical University Cancer Institute and Hospital from 2011 to 2015. Preoperative evaluation confirmed the presence of resectable pancreatic cancer. The median age of patients was 60 (range from 42-80). There were 65 cases (63.1%) of tumor located in the head and uncinate process of pancreas, and 38 cases (36.9%) were located in the pancreatic body and tail. Based on the pathological TNM staging system, 43 patients (41.7%) were in Stage I and 60 patients (58.3%) were in Stage II. There were 63 patients (61.2%) with tumor size smaller than 4 cm and 40 patients (38.8%) with size greater than 4 cm. A total of 28 cases (27.2%) exhibited local lymph node metastasis. In addition, there were 18 cases (17.5%) with presence of carcinoma cell emboli, 40 cases (38.8%) with perineural invasion, 81 cases (78.6%) with pancreatic capsule invasion, and 23 cases (22.3%) with vascular invasion. The general characteristics, including laboratory tests, are summarized in Table 1.

### Independent prognosis factors for resectable PDAC with stage I and II

We initially selected 28 risk factors that may affect the overall survival of patients with PDAC after radical surgery based on basic clinical information, pathological finding and laboratory results of patients. Univariate COX analysis identified 7 candidate indicators for poor prognosis, including age (HR=0.687, p=0.195), preoperative CA19-9 (HR=1.454, p=0.195), preoperative CEA (HR=2.348, p=0.001), preoperative DBIL (HR=0.654, p=0.107), postoperative CA19-9 (HR=1.636, p=0.025), postoperative CEA (HR=2.187, p=0.012), and postoperative CA24-2 (HR=1.849, p=0.007), which were further evaluated by multivariate COX regression analysis. From that, the forward and backward LR methods verified that elevated preoperative CEA (HR=2.212, p=0.002, p<0.05) and postoperative CA24-2 (HR=1.731, p=0.017, p<0.05) were independent factors of poor prognosis and OS (Table 2).

### Preoperative CEA and postoperative CA24-2 levels correlated with clinical characteristics

We compared inter-group relationship between preoperative CEA, postoperative CA24-2, and clinical pathological features. Elevation of either preoperative CEA or postoperative CA24-2 was given a score of 1. Consequently, patients were divided into three groups with scores of 0 (no elevation), 1 (elevation of either CEA or CA24-2), 2 (elevation of both CEA and CA24-2). Results showed that late stage PDAC was highly correlated with increased preoperative CEA, postoperative CA24-2 and the combination of two (p=0.027, p=0.008 and p=0.004). Regional lymph node invasion also correlated with postoperative CA24-2 and the combination of two (p=0.003, p=0.002). Interestingly, primary tumor (T) was only correlated with the combination of both elevated CEA and CA24-2 (p=0.035) (Table 3).

### Combined scores of postoperative CA24-2 and preoperative CEA were associated with postoperative prognosis of PDAC

**Figure 1:**
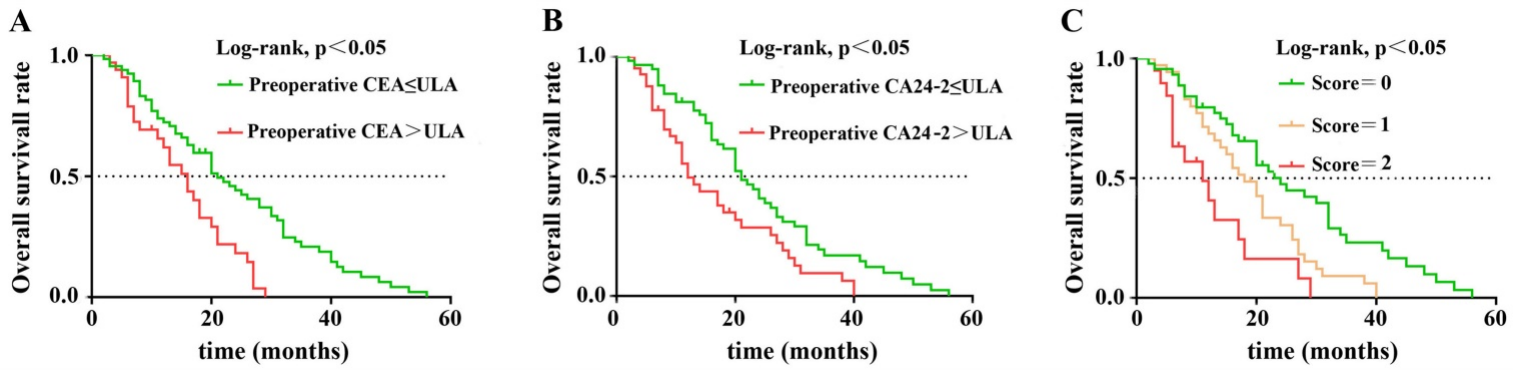
Kaplan-Meier overall survival curves for 103 patients with resectable PDAC stratified by (**A**) preoperative CEA level, (**B**) postoperative CA24-2 level, (**C**) combined score of preoperative CEA and postoperative CA24-2 levels.

In nonparametric tests, we found that patients with either elevated preoperative CEA or postoperative CA24-2 predicted a lower OS after surgery (21 vs. 16 months, p=0.001; 21 vs. 12 months, p=0.005) When preoperative CEA and postoperative CA24-2 were combined, patient OS decreased further (p<0.001) suggesting an increase in both factors were more detrimental to patient survival. The Kaplan-Meier method was used to compare the effects of preoperative CEA and postoperative CA24-2 on the OS between groups (Figure 1 A and B). The combined scores of preoperative CEA and postoperative CA24-2 embodied a stronger correlation with OS in which patients had lower OS compared to patients with an increase of either preoperative CEA or postoperative CA24-2 (11 vs. 21 vs. 16 months, p<0.001).

## Discussion

In our clinical work, we found that patients with resectable PDAC often have different prognosis after radical resection. Grading of PDAC and surgical guideline are primarily based on imaging results and tumor anatomy. Compared to other cancers with established biomarkers, such as PR, ER and HER-2 in breast cancer 12 and RAS, BRAF, MMR in colon cancer 13, PDAC lacks effective markers to assist in diagnosis, treatment and prognosis. Therefore, this study analyzed clinical data, laboratory tests and pathological diagnosis before and after surgical resection to determine factors that may affect the prognosis of PDAC patients. The study evaluated 29 prognostic factors derived from basic clinical information, pathological finding and laboratory results. All parameters were analyzed by univariate and multivariate COX proportional-hazards model. Information such as age, gender, tumor location and TNM staging were common factors used in analysis of tumor development and prognosis. In this study, we grouped primary tumors into two categories with a cutoff value of 4 cm. This classification has been demonstrated to resemble more closely with prognostic outcome 14. We also performed analysis of tumors categorized into three groups, and the results did not differ from that of the two categories. In addition, histopathology such as neurological invasion, vascular invasion, carcinoma cell embolism and pancreatic capsule invasion were also included in this study 5, 15. Laboratory indicators were selected based on convenience and ease of collection. They are commonly used to evaluate patient condition before and after surgery. CA19-9, CEA and CA24-2 are tumor markers that reflect PDAC progression from tumorigenesis, local progression to metastasis 16. For this reason, tumor markers were collected preoperatively and 1 month after surgery. Serum enzymes such as LDH and ALP were shown to be associated with prognosis in PDAC 17. Bilirubin and ALB are important indicators of liver metabolism and nutritional status of the patient, which may also influence prognosis 18. Recent studies have found that preoperative NLR affects prognosis of patients undergoing radical surgery, but there is a lack of reference NLR level 19. In our study, multivariate COX regression analysis showed that OS in patients that underwent radical resection during early PDAC (stage I and II) had no significant correlation with age, gender, tumor location and TNM stage. There was also no significant correlation between prognosis and the presence of neurological invasion, vascular invasion, vascular tumor thrombus and pancreatic capsule invasion. From serum markers, we found that preoperative CEA and postoperative CA24-2 were independent risk factors of radical resection of PDAC. Other markers, such as CA19-9, LDH, ALD, TBIL, DBIL, ALB, and NLR did not show strong correlation with OS in early stage PDAC patients. It should be noted that a meta-analysis of NLR in PDAC patients revealed no clear cutoff value for NLR, but a cutoff value of 5 was sufficiently stable to evaluate postoperative prognosis 19, 20. Therefore, this study used NLR=5 as the cutoff value, and found no statistical difference between NLR=2.3^21^, NLR=3 22, NLR=4 23 and NLR=5 on OS.

CA19-9 has been touted as a reliable serum marker for PDAC because of its high sensitivity of 70%-90% and specificity of 90% for diagnosis 24. However, in this study CA19-9 did not correlate with postoperative survival of PDAC patients. In addition, due to a lack of Lewis antigen in 6.9% of patients, the sensitivity of CA19-9 in our test population was only 80%. This phenomenon limited the ability of CA19-9 as an independent biomarker for PDAC prognosis 25. Patients with resectable PDAC in our study were all in stage I and stage II, and accuracy of CA19-9 can vary with disease stage 26. Furthermore, one study indicated that the different cutoff values of CA19-9 may have different prognostic efficacy. Therefore, in early stages, CA19-9 may not be an effective biomarker compared to late stages of PDAC 27.

Our study found that preoperative CEA and postoperative CA24-2 were independent markers for poor prognosis of radical PDAC resection. CEA is the second most common serum biomarker used to diagnose PDAC. A meta-analysis estimated that the average sensitivity of CEA for PDAC detection was 44.2% (95%CI, 38.5%-50.0%) with an average specificity of 87.5% (95%CI, 82.5%-91.2%) 28. While some suggested that CEA is inferior to CA19-9 in identifying PDAC, the specificity of the two are similar. A later study supported CEA as a potential biomarker in Lewis-negative PDAC patients, especially in stage I and stage II with better correlation to prognosis 29. This was consistent with the results of our study. We found that low preoperative CEA correlated with longer survival in PDAC patients after radical surgery. Another marker, CA24-2, is used in conjunction with CA19-9 in early diagnosis of PDAC. Although CA24-2 was found to be less sensitive than CA19-9 in diagnosing PDAC, it was more specific 30. One study found that pancreatic tumor size of more than 4cm showed significantly higher serum CA24-2 level, and cancer invasiveness also correlated positively with CA24-2 level 31. Interestingly, our study found that postoperative CA24-2 level correlated better with tumor size, lymph node metastasis and PDAC stage than preoperative CA24-2. Our study supported the notion that postoperative tumor markers play an important role in resectable PDAC prognosis. When patients showed elevations in both preoperative CEA and postoperative CA24-2 markers, we found that they had lower OS compared with each biomarker alone. This observation suggested that patients with an increase in the combined index will have a worse prognosis. Therefore, combination biomarkers should be used to improve prognostic power.

The current study has several limitations. First, our study evaluated biomarkers for prognosis in the context of early stage PDAC. Established prognostic factors such as CA19-9 31, LDH, ALP 18 and NLR 20 were mostly used for later stage PDCA and distant metastasis, which was not found to be significant for in ours. It should also be noted that recruited patients were not refrained from receiving other treatments after the initial chemotherapy from our study. Possible treatment variability may have affected the average OS of patients in this study. Postoperative adjuvant therapy therefore should be better controlled in further studies.

## Conclusion

Our study revealed that preoperative CEA and postoperative CA24-2 are biomarkers for prognosis of patients in stage I and II PDAC that underwent radical resection. Elevated CEA and CA24-2 independently and in combination predicted poor survival of patients, where the combination of the two led to worse prognosis. In order to determine the prognosis of patients, tumor markers from before and after surgery should be combined to accurately manage the timing of surgery and adjuvant therapy. A multicenter prospective cohort will be established to investigate the relationship between prognosis and a more comprehensive array of biomarkers in patients at different stages of PDAC undergoing radical surgery.

## Figures and Tables

**Table 1:** Clinicopathologic characteristics of patients with resectable PDAC

Characteristics	Number	Percentage (%)	Median OS (months)	p-value
**Total**	103	100	20	
**Clinical characteristics**				
**Age (years)**				
Median (range) 60 (42-80)				
<65 vs. ≥65	81 vs. 22	78.6 vs. 21.4	20 vs. 25	0.183
**Gender** (Male vs. Female)	65 vs. 38	63.1 vs. 36.9	18 vs. 21	0.255
**Tumor location** (Head and Uncinate process vs. Body and Tail)	65 vs. 38	63.1 vs. 36.9	18 vs. 20	0.393
**Stage** (Stage Ⅰ vs. Stage Ⅱ)	43 vs.60	41.7 vs. 58.3	20 vs. 18	0.327
**Primary tumor(T)** (1 and 2 vs. 3)	63 vs. 40	61.2 vs. 38.8	20 vs. 18	0.960
**Regional lymph(N)** (0 vs. 1)	75 vs. 28	72.8 vs. 27.2	20 vs. 20	0.337
**Histopathology characteristics**				
**Carcinoma cell embolus** (Present vs. Absent)	18 vs. 85	17.5 vs. 82.5	21 vs. 20	0.332
**Perineural invasion** (Present vs. Absent)	40 vs. 63	38.8 vs. 61.2	17 vs. 20	0.526
**Pancreatic capsule invasion** (Present vs. Absent)	81 vs. 22	78.6 vs. 21.4	20 vs. 17	0.480
**Vascular invasion** (Present vs. Absent)	23 vs. 80	22.3 vs. 77.7	22 vs. 18	0.232
**Laboratory testing**				
**Preoperative CA19-9** (≤ULN vs. >ULN)	18 vs. 85	17.5 vs. 82.5	21 vs. 18	0.182
**Preoperative CEA** (≤ULN vs. >ULN)	69 vs. 34	67.0 vs. 33.0	21 vs. 16	<0.001
**Preoperative CA24-2** (≤ULN vs. >ULN)	39 vs. 64	37.9 vs. 62.1	21 vs. 18	0.332
**Preoperative LDH** (≤ULN vs. >ULN)	83 vs. 20	80.6 vs. 19.4	18 vs. 24	0.446
**Preoperative ALP** (≤ULN vs. >ULN)	46 vs. 57	44.7 vs. 55.3	20 vs. 20	0.650
**Preoperative TBIL** (≤ULN vs. >ULN)	44 vs. 59	42.7 vs. 57.3	18 vs. 20	0.696
**Preoperative DBIL** (≤ULN vs. >ULN)	27 vs. 76	26.2 vs. 73.8	17 vs. 21	0.097
**Preoperative Albumin** (≥LLN vs. <LLN)	95 vs. 8	92.2 vs. 7.8	22 vs. 18	0.232
**Preoperative NLR** (≤5 vs. >5)	93 vs. 10	90.3 vs. 9.7	20 vs. 20	0.289
**Postoperative CA19-9** (≤ULN vs. >ULN)	58 vs. 45	56.3 vs. 43.7	21 vs. 13	0.021
**Postoperative CEA** (≤ULN vs. >ULN)	89 vs. 14	86.4 vs. 13.6	20 vs. 12	0.008
**Postoperative CA24-2** (≤ULN vs. >ULN)	61 vs. 42	59.2 vs. 40.8	21 vs. 12	0.005
**Postoperative Albumin** (≥LLN vs. <LLN)	52 vs. 51	50.5 vs. 49.5	20 vs. 17	0.361

**Table 2:** Univariate and multivariate analysis of prognostic factors

Predictor	Patients (n)	HR (95%CI)	p-value
**Univariate analysis**			
**Age** (<65 vs. ≥65)	81 vs. 22	0.687(0.389-1.213)	0.195
**Gender** (male vs. female)	65 vs. 38	0.776(0.496-1.215)	0.268
**Tumor location** (head and uncinate process vs. body and tail)	65 vs. 38	0.826(0.528-1.294)	0.404
**Stage** (stage Ⅰ vs. stage Ⅱ)	43 vs. 60	1.236(0.801-1.909)	0.339
**Primary tumor(T)** (1 and 2 vs. 3)	63 vs. 40	1.011.(0.654-1.562)	0.961
**Regional lymph(N)** (0 vs. 1)	75 vs. 28	1.264(0.773-2.066)	0.350
**Carcinoma cell embolus** (present vs. absent)	18 vs. 85	0.765(0.439-1.322)	0.344
**Perineural invasion** (present vs. absent)	40 vs. 63	1.149(0.741-1.782)	0.535
**Pancreatic capsule invasion** (present vs. absent)	81 vs. 22	0.834(0.499-1.395)	0.490
**Vascular invasion** (present vs. absent)	23 vs 80	0.740(0.446-1.228)	0.244
**Preoperative CA19-9** (≤ULN vs. >ULN)	18 vs. 85	1.454(0.826-2.561)	0.195
**Preoperative CEA** (≤ULN vs. >ULN)	69 vs. 34	2.348(1.437-3.835)	0.001
**Preoperative CA24-2** (≤ULN vs. >ULN)	39 vs. 64	1.236(0.797-1.919)	0.344
**Preoperative LDH** (≤ULN vs. >ULN)	83 vs. 20	0.813(0.471-1.403)	0.457
**Preoperative ALP** (≤ULN vs. >ULN)	46 vs. 57	1.102(0.716-1.696)	0.658
**Preoperative TBIL** (≤ULN vs. >ULN)	44 vs. 59	1.088(0.705-1.680)	0.702
**Preoperative DBIL** (≤ULN vs. >ULN)	27 vs. 76	0.654(0.390-1.096)	0.107
**Preoperative Albumin** (≥LLN vs. <LLN)	95 vs. 8	1.126(0.517-2.452)	0.765
**Preoperative NLR** (≤5 vs. >5)	93 vs. 10	1.513(0.689-3.320)	0.302
**Postoperative CA19-9** (≤ULN vs. >ULN)	58 vs. 45	1.636(1.064-2.517)	0.025
**Postoperative CEA** (≤ULN vs. >ULN)	89 vs. 14	2.187(1.192-4.014)	0.012
**Postoperative CA24-2** (≤ULN vs. >ULN)	61 vs. 42	1.849(1.183-2.891)	0.007
**Postoperative Albumin** (≥LLN vs. <LLN)	52 vs 51	1.216(0.791-1.871)	0.373
**Multivariate analysis**			
**Preoperative CEA** (≤ULN vs. >ULN)	69 vs. 34	2.212(1.351-3.623)	0.002
**Postoperative CA24-2** (≤ULN vs. >ULN)	61 vs. 42	1.731(1.103-2.717)	0.017

**Table 3:** Correlation between preoperative CEA/postoperative CA24-2 and clinicopathologic characteristics

Characteristics	Value	Preoperative CEA			Postoperative CA24-2			Combination of 2 factors			
		≤ULN	>ULN	p-value	≤ULN	>ULN	p-value	0	1	2	p-value
Age (years)	<65	57	24	0.162	49	32	0.615	39	28	14	0.499
	≥65	12	10		12	10		8	8	6	
Gender	Male	41	24	0.269	39	28	0.534	26	26	13	0.281
	Female	28	10		24	14		21	10	7	
Tumor location	Head and Uncinate process	41	24	0.269	42	23	0.145	31	21	13	0.761
	Body and Tail	28	10		19	19		16	15	7	
Primary tumor(T)	1 and 2	44	19	0.440	39	24	0.487	28	27	8	0.035
	3	25	15		22	18		19	9	12	
Regional lymph(N)	0	55	20	0.025	51	24	0.003	42	22	11	0.002
	1	14	14		10	18		5	14	9	
Stage	Ⅰ	34	9	0.027	32	11	0.008	25	16	2	0.004
	Ⅱ	35	25		29	31		22	20	18	
Carcinoma cell embolus	Present	14	4	0.284	11	7	0.858	9	7	2	0.618
	Absent	55	30		50	35		38	29	18	
Perineural invasion	Present	27	13	0.930	25	15	0.590	18	16	6	0.566
	Absent	42	21		36	27		29	20	14	
Pancreatic capsule invasion	Present	52	29	0.247	47	34	0.635	36	27	18	0.380
	Absent	17	5		14	8		11	9	2	
Vascular invasion	Present	17	6	0.423	47	33	0.855	11	9	3	0.671
	Absent	52	28		14	9		36	27	17	

## Abbreviations

LDH: lactate dehydrogenase
CA19-9: carbohydrate antigen 19-9
CA24-2: carbohydrate antigen 24-2
CA12-5: carbohydrate antigen 12-5
ALB: albumin
PDAC: pancreatic ductal adenocarcinoma
OS: overall survival
ALP: alkaline phosphatase
CEA: carcinoembryonic antigen
ULN: upper limit of normal
LLN: lower limit of normal
HR: hazard ratio
TBIL: total bilirubin
DBIL: direct bilirubin
DSS: disease-specific survival
DFS: disease-free survival
NLR: neutrophils/lymphocytes ratio
CRP: C-reactive protein
